# Synthesis of Two Porous CdS Rods by Anion Exchange Method and Their Photocatalytic Properties

**DOI:** 10.3390/nano12183190

**Published:** 2022-09-14

**Authors:** Liwei Wang, Ming Meng, Ruirui Zheng, Xiaoli Li, Honglei Yuan

**Affiliations:** School of Physics and Telecommunications Engineering, Zhoukou Normal University, Zhoukou 466001, China

**Keywords:** CdS, hollow structure, anion exchange method, photocatalytic activity

## Abstract

Semiconductor materials with pore structure have excellent physicochemical properties for photocatalytic reactions. Here, the one-step vulcanization of Cd-based MOF solid rods was successfully developed to synthesize two kinds of CdS rods with pore structure: hollow rods (HRs) and mesoporous rods (MRs). Among the three catalysts, the CdS HRs showed the highest photocatalytic efficiency, which could remove about 96.0% of RhB in 30 min under visible light irradiation. The enhanced photocatalytic activity of CdS HRs benefits from its novel hollow structure, which enhances the visible light absorption capability and the separation efficiency of photogenerated electron–hole pairs. The successful synthesis of CdS HRs has guiding significance for the design and synthesis of other hollow structures with high photocatalytic activity.

## 1. Introduction

As one of the potential strategies to deal with environmental pollution and the energy crisis, semiconductor photocatalysis technology has received extensive attention worldwide [1,2,3,4,5]. Among the semiconductor photocatalysts, CdS is one of the most extensively studied materials because of its suitable energy-band structure, abundant morphologies, and excellent carrier mobility [6,7,8]. However, the practical application of CdS still has low photocatalytic activity due to severe photocorrosion and photogenerated carrier recombination under visible light. Structural engineering is an effective strategy to enhance the physicochemical properties of semiconductor photocatalysts [9,10,11,12]. Nanomaterials with pores are good candidates due to several unique advantages, including their low density, strong light capture capability, and large surface area [13,14,15,16]. As an important photocatalyst member, CdS with porous nanostructure is required to improve the photocatalytic activity.

Nanoparticles with complex structures are not easy to synthesize, and the design and synthesis of porous nanostructures has become an important research field in the frontiers of chemistry and materials science [17,18,19,20]. Recently, ion exchange has been developed as an efficient strategy for the fabrication of hollow and mesoporous structures. Yu et al. successfully synthesized CdS nanoflowers with mesoporous structure via anion exchange method under hydrothermal conditions, using Cd(OH)_2_ and Na_2_S as precursors [21]. Lou et al. developed a multistep method combining solution growth, sulfidation, and cation exchange processes for the synthesis of novel CdS multicavity hollow particles, which showed high performance in photocatalytic CO_2_ reduction [22]. They also developed a two-step sulfidation method based on cadmium Prussian blue analogs to synthesize CdS particles with a “frame-cage” bilayer structure, and the obtained CdS particles exhibited high photocatalytic water-splitting activity [23]. Shi et al. successfully transformed Cd(OH)Cl nanorods into CdS/ZnS/PdS NTs through a one-step vulcanization progress for the first time, in the presence of ZnCl_2_ and Na_2_PdCl_4_ [24]. In this context, there is still an imminent need to develop a simple method to construct CdS hollow nanostructures.

Herein, CdS hollow rods (HRs) and mesoporous rods (MRs) were successfully synthesized through an anion exchange strategy. The morphology, structure, and composition of the samples before and after vulcanization were characterized by SEM, TEM, XRD, and XPS, which confirmed the conversion of the Cd-Cys solid rods (SRs) to CdS HRs. Compared with CdS solid spheres (SSs) and CdS MRs, CdS HRs exhibited excellent photocatalytic activity when degrading RhB under visible light.

## 2. Materials and Methods

### 2.1. Synthesis of Cd-Cys SRs

In a typical synthesis, 1 mmol of Cd(NO_3_)_2_ and 2 mmol of cysteine were added to 50 mL of ultrapure water and stirred for 30 min. Then, the pH of the mixture was adjusted to 12 with ammonia. The resulting solution was heated to boiling and maintained for 300 min.

### 2.2. Preparation of CdS HRs

A total of 15 mg of Cd-Cys SRs and 1 mmol of thioacetamide (TAA) were dispersed in 30 mL of absolute ethanol, and stirred for 30 min. The mixture was transferred into a Teflon-lined stainless-steel autoclave. After sealing, the autoclave was heated to 130 °C for 6 h. The products were washed with ethanol three times to remove ions and impurities.

### 2.3. Preparation of CdS MRs

A total of 15 mg of Cd-Cys SRs and 1 mmol of Na_2_S were added into 30 mL of ultrapure water, and stirred for 30 min. The mixture was then heated to 130 °C in autoclave for 6 h. The products were washed with deionized water and ethanol several times to remove ions and impurities.

### 2.4. Characterizations

The surface and morphology images of samples were measured with a field-emission scanning electron microscope (FESEM, Zeiss sigma 300, Jena, Germany). Microstructures of the samples were tested with a transmission electron microscope (TEM, FEI Talos F200, FEI, Saugus, MA, USA) and the operating voltage was 200 kV. The X-ray diffraction (XRD, Bruker, Germany) patterns of the samples were carried out on a Bruker D8 advanced diffractometer. The UV–vis absorption spectra of samples were performed on a PerkinElmer Lambda 35 photometer. The elemental compositions and chemical states of the samples were obtained by X-ray photoelectron spectra (XPS) on a Thermo Scientific (ESCALAB, 250Xi, Thermo, Waltham, MA, USA) with a monochromatic Al Ka excitation source.

### 2.5. Photocurrent Measurement

The photocurrent measurements for samples were performed in the electrochemical workstation (CHI660E) under white-light irradiation (xenon lamp, 300 W, Chenhua, China). A total of 5 mg of the sample was dispersed in 1 mL of ethanol, then drop-coated on ITO glass and dried at 60 °C. Samples on indium tin oxide substrate were used as the working electrode in the three-electrode system; Pt foil and Ag/AgCl were used as counter electrode and reference electrode, respectively. In addition, 0.5 M of Na_2_SO_4_ aqueous solution was used as the electrolyte.

### 2.6. Photocatalytic Activity Measurement

The optical system used for photocatalytic reaction consisted of xenon lamp (300 W) and a band-pass filter (with the bandwidth of 400–780 nm) that ensured the irradiation in visible range. CdS SSs, CdS HRs, and CdS MRs were used for photodegradation of RhB solution. Typically, 6 mg sample was added into 20 mL RhB solution (1.0 × 10^−5^ M) and stirred for 30 min in the dark to achieve adsorption equilibrium. The UV–vis absorption spectra of filtrates every 10 min of illumination were recorded to analyze photocatalytic activity.

## 3. Results and Discussion

**Figure 1:**
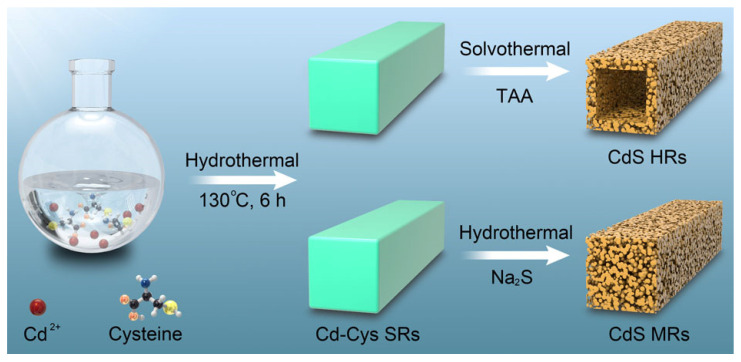
Schematic illustration of the synthesis of CdS HRs and CdS MRs.

The typical synthesis of CdS HRs and MRs is depicted in Figure 1. The Cd-Cys SRs were firstly synthesized through a hydrothermal method. Then, the Cd-Cys solid precursors were transformed into CdS HRs through a solution-sulfidation process under solvothermal conditions. The inward-diffused S^2−^ from the decomposition of TAA and the faster outward-diffused Cd^2+^ from the surface of the Cd-Cys rods were combined to form CdS crystals. With the progress of the anion exchange reaction, the CdS hollow rods were formed [25,26]. When Cd-Cys SRs reacted with Na_2_S in a hydrothermal environment, CdS mesoporous rods were formed, due to the faster release of S^2−^ from Na_2_S.

**Figure 2:**
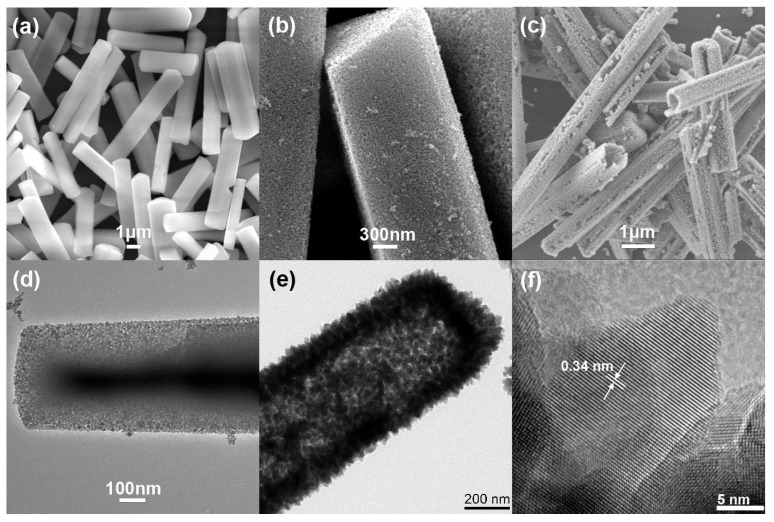
(**a**–**c**) SEM images of samples; (**d**,**e**) TEM images of CdS MRs and CdS HRs; (**f**) HRTEM image of CdS HRs.

FESEM and TEM were adopted to monitor the evolution of the surface, morphology, and microstructure of the samples. It can be seen from Figure 2a that the Cd-Cys SRs are uniform and the surface of the rod is very smooth. After vulcanization, the Cd-Cys SRs are transformed into the CdS MRs and CdS HRs. As shown in Figure 2b, the surface of CdS MRs is composed of small-sized CdS nanoparticles. As can be seen from Figure 2c, the surface of CdS HRs is composed of CdS nanoparticles with pores on them. There are distinct cavities at the ends of the broken rods. Figure 2d shows the CdS MRs formed by connecting fine CdS nanoparticles (about 10 nm) with tiny gaps between them. Figure 2e shows that the surface of the CdS HRs is composed of rice-like CdS nanoparticles (about 40 nm) and that the internal cavity is obvious. During the vulcanization reaction, there is an obvious gap between the CdS shell and Cd-Cys core, forming a unique core–shell nanostructure in the structural intermediate (see Supplementary Materials Figure S1). In the high-resolution TEM image (Figure 2f), the surface nanoparticles are very crystalline and the lattice spacing is 0.34 nm, corresponding well to the (002) crystal plane of the wurtzite CdS crystal. To better reveal the advantages of the CdS HRs, CdS solid spheres (SSs) were also synthesized as counterparts (see Supplementary Materials Figure S2).

**Figure 3:**
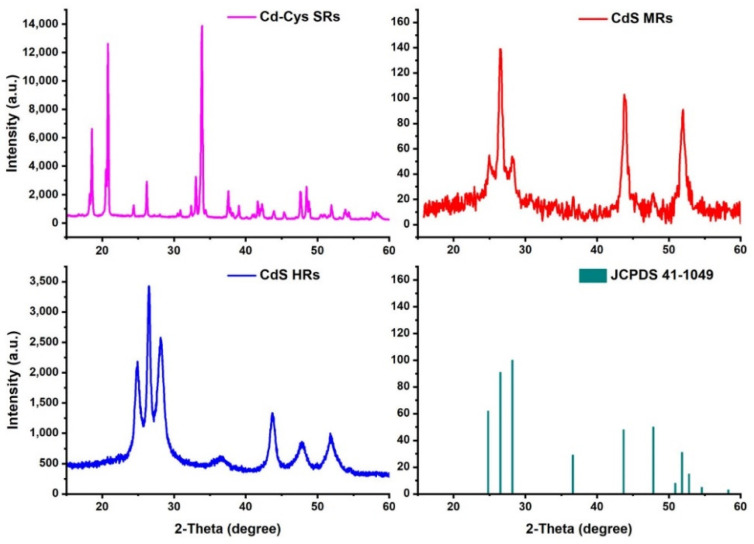
XRD patterns of samples.

The crystallographic structure and phase composition of as-prepared samples were further characterized by XRD pattern, shown in Figure 3. All the diffraction peaks of Cd-Cys SRs belong to the orthorhombic crystal system [27]. After vulcanization, all the XRD peaks pointed to the hexagonal wurtzite CdS crystals (JCPDS 41-1049). The XRD results indicate a complete phase transformation from Cd-Cys SRs to CdS HRs. The XRD pattern also indicated that the MRs were wurtzite CdS crystals. However, the crystallinity was not as good as that of CdS HRs.

**Figure 4:**
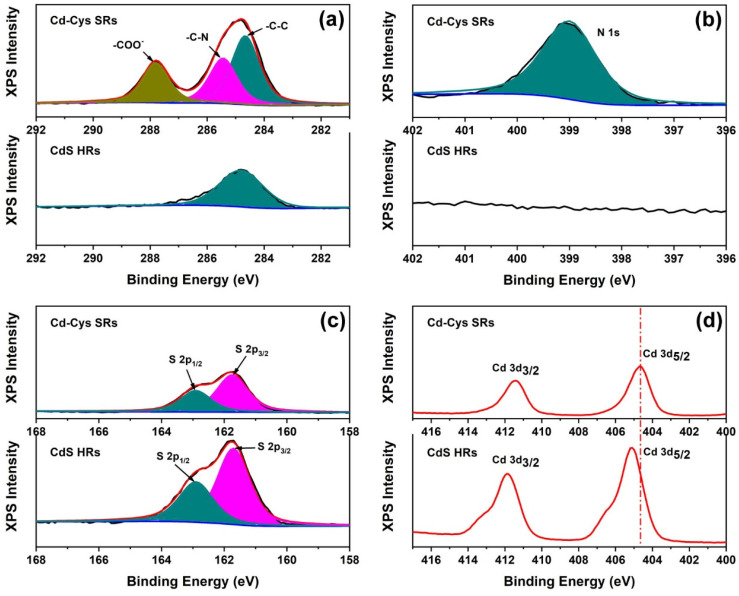
XPS patterns of C 1s (**a**), N 1s (**b**), S 2p (**c**), and Cd 3d (**d**) in samples before and after vulcanization.

To clarify the elemental compositions and chemical states, XPS spectroscopy was carried out on samples before and after vulcanization. The high-resolution XPS spectra of C 1s, N 1s, S 2p and Cd 3d in Cd-Cys SRs and CdS HRs are shown in Figure 4. Before vulcanization, the binding energy of C 1s in Cd-Cys SRs was located at 284.6, 285.5, and 287.8 eV (Figure 4a), which was attributed to the -C-C, -C-N, and -COO- structures, respectively [28]. The N1s was located at 399 eV resulted from the -NH_2_ of cysteine. In the high-resolution S 2p spectra shown in Figure 4c, the two characteristic peaks located at 161.7 eV and 162.9 eV were assigned to S 2p_3/2_ and S 2p_1/2_, respectively. The peaks at 404.7 and 411.4 eV in Figure 4d could be assigned to the binding energies of Cd 3d_5/2_ and Cd 3d_3/2_, respectively. After vulcanization, the XPS spectra of the -C-N, -COO- and -NH_2_ in the cysteine molecule disappeared. The binding energy of Cd 3d_5/2_ and Cd 3d_3/2_ in CdS HRs was slightly higher than that in Cd-Cys SRs, which can be explained by the transfer of electrons around Cd to S in CdS HRs [29]. The XPS results confirmed the elemental changes during the conversion of Cd-Cys SSs to CdS HRs. Therefore, the formation of CdS HRs is an anion exchange process, i.e., S^−^ replaces cysteine radicals.

**Figure 5:**
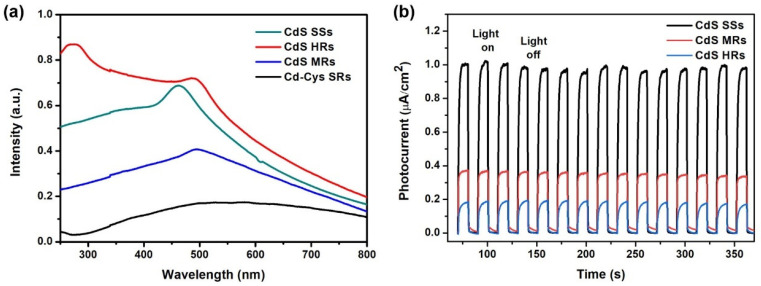
(**a**) UV–vis absorption spectra and (**b**) photocurrent responses of samples.

The optical properties of the samples are shown in Figure 5a. Cd-Cys SRs are essentially nonabsorbing in the visible region. The UV–vis spectrum of the CdS HRs shows a slightly redshifted absorption edge compared with the other samples. This result indicates that the CdS HRs possess a reduced bandgap as reflected by the Tauc plots and can utilize a larger fraction of the solar spectrum (Figure S3, Supplementary Materials). CdS HRs have strong light absorption properties and a wide visible-light absorption range, which may be related to their larger size and hollow structure [30]. However, the absorption intensity of CdS SSs and CdS MRs decreases in the short-wavelength region of visible light, which may be related to their solid internal structure. Photocurrent response analysis has been widely recognized as an effective means to evaluate the separation capability of photogenerated carriers. A higher photocurrent response indicates better charge separation and migration performance. The transient photocurrent responses of samples under visible light are shown in Figure 5b. CdS SSs have the highest photocurrent intensity, followed by CdS MRs, and the weakest by CdS HRs. This result reveals that photogenerated carriers travel faster within a single crystal than between nanoparticles [31,32]. Many charge carriers are generated in CdS HRs, which cannot reach the ITO surface due to steric hindrance. However, this may facilitate the rapid diffusion of photogenerated carriers to the surface to participate in redox reactions.

**Figure 6:**
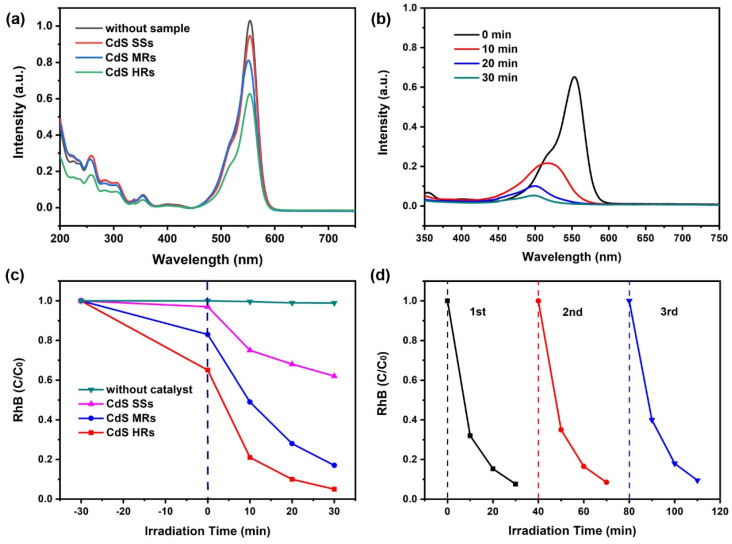
(**a**) Adsorption properties of samples; (**b**) the degradation curves of CdS HRs under visible light for 30 min; (**c**) the degradation rate of RhB by different samples; (**d**) the repeatability tests studied on the CdS HRs for three recycles.

The specific surface area of the sample is an important factor affecting the photocatalytic activity. The specific surface area of CdS HRs was 105.7 m^2^/g, and that of CdS MRs was 86.3 m^2^/g. The large specific surface area of CdS HRs may originate from the low density of the hollow structure (Figure S4, Supplementary Materials). The adsorption capacity of the samples to organic molecules was obtained by UV–vis absorption spectroscopy. Figure 6a showed that the absorption spectra of RhB solution before and after mixing with CdS SSs, CdS HRs, and CdS MRs, respectively. The adsorption ratios of RhB on CdS SSs, CdS MRs, and CdS HRs were 4%, 17%, and 35%, respectively. Therefore, CdS HRs have the strongest adsorption capacity of RhB molecules, which is related to its open hollow structure. The photocatalytic properties of CdS HRs were examined under visible-light irradiation. In the CdS HRs photocatalytic system, the concentration of RhB solution decreased rapidly with the illumination time, as shown in Figure 6b. As counterparts, the photocatalytic activity of CdS SSs and CdS MRs were also surveyed under the same conditions. Figure 6c shows that the normalized concentration changes in RhB solution under different photocatalysts. The photocatalytic activity of CdS HRs is significantly higher than that of CdS counterparts. After three consecutive photocatalytic tests, the CdS HRs still maintained high photocatalytic activity, as shown in Figure 6d, indicating the high stability of CdS HRs. Moreover, the crystalline structure of the CdS HRs is almost unchanged after the cycling tests, indicating the high stability of the photocatalyst (Figure S5a, Supplementary Materials). The hollow structure is also maintained after the cycling tests (Figure S5b, Supplementary Materials), which reveals the structural robustness of the sample.

Under visible-light irradiation, the electrons on the valence band of CdS transition to the conduction band. Subsequently, the photogenerated electrons and holes are separated and diffused to the catalyst surface, where they encounter RhB molecules and cause the RhB molecules to be decomposed into CO_2_ and H_2_O. The enhanced photocatalytic performance of CdS HRs may originate from their open hollow structure. The cavity inside the CdS HRs allows multiple reflections of incident light, which favors the light absorption ability to generate more charge carriers [33,34]. Moreover, the large specific surface area of the CdS HRs provides abundant active sites and accelerates the photocatalytic reaction. In addition, the thin shell layer also helps the photogenerated carriers to quickly diffuse to the surface to participate in the reaction, in turn consuming the photogenerated carriers in time, increasing the photocatalytic stability of CdS HRs. In CdS MRs, photogenerated carriers can shuttle between nanoparticles, but micropores can increase the resistance to the diffusion of guest molecules in the host material, which has a detrimental effect on chemical reactions involving macromolecules or in viscous systems.

## 4. Conclusions

In conclusion, CdS HRs and MRs were successfully synthesized by a simple and efficient anion exchange method. XRD patterns and SEM and TEM images showed that the samples had regular morphology and structure. XPS data confirmed that the synthesis of CdS HRs was an anion exchange process. Compared with CdS SSs and CdS MRs, CdS HRs exhibited the highest photocatalytic activity under visible light irradiation due to the structural advantages. The cavity inside the CdS HR allows for multiple reflections of incident light, enhancing visible light absorption. The thin-shelled topologies would reduce the distance for the transport of charge carriers. More importantly, the large specific surface areas of hollow structure can provide abundant active sites for redox reactions. The developed synthetic strategy may provide new inspirations for the design and construction of high-performance photocatalysts with advanced structures.

## Data Availability

Not applicable.

## Supplementary Materials

The following supporting information can be downloaded at: https://www.mdpi.com/article/10.3390/nano12183190/s1, Figure S1: TEM image of Cd-Cys@CdS core–shell rods. Figure S2: SEM image of CdS SSs. Figure S3: Tauc plots of the CdS HRs. Figure S4: N_2_ adsorption–desorption isotherms for CdS MRs and CdS HRs. Figure S5: (a) XRD pattern and (b) TEM image of the CdS HRs after the cycling tests.
