# Supplementary material for: Synthesis of Two Porous CdS Rods by Anion Exchange Method and Their Photocatalytic Properties
Source: Nanomaterials (Basel). 2022 Sep 14;12(18):3190. doi: 10.3390/nano12183190 (PMC9505071; doi:10.3390/nano12183190)
Supplement: Supplementary file 1 [file nanomaterials-12-03190-s001.zip › nanomaterials-1892336-supplementary.pdf]

Electronic Supplementary Material

Synthesis of two porous CdS rods by anion exchange method and their photocatalytic properties

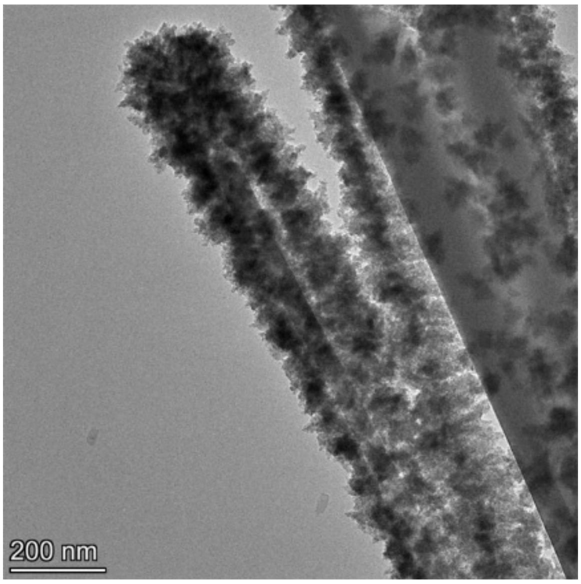

Figure S1: TEM image of Cd-Cys@CdS core-shell rods.

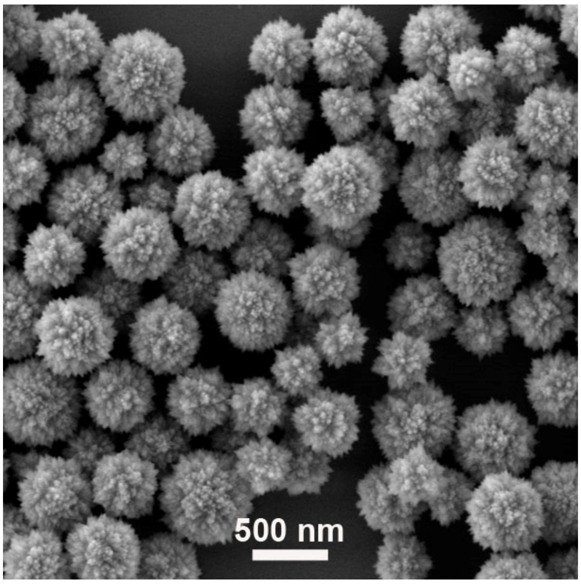

Figure S2: SEM image of CdS SSs. Typically, 1 mmol $Cd(NO_3)_2$ and 2 mmol cysteine were dissolved in 30 mL deionized water to form a clear solution after stirring for 30 min. The solution was then transferred into a 50 mL autoclave. The autoclave was sealed and heated to 130 ℃ for 6 h and cooled naturally to room temperature.

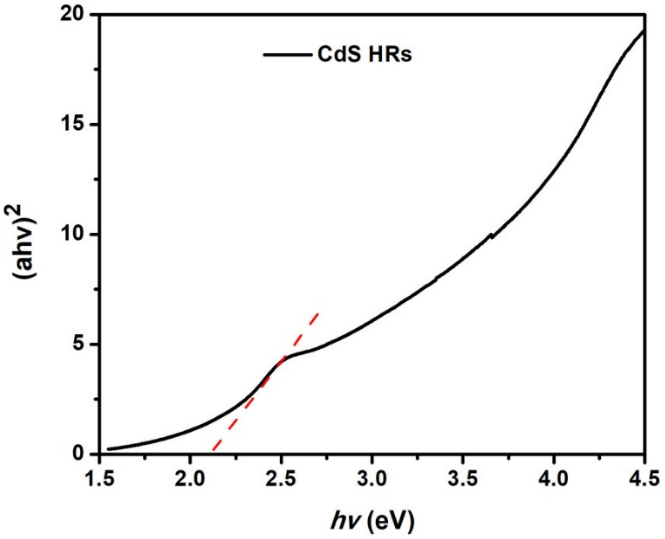

Figure S3: Tauc plots of the CdS HRs.

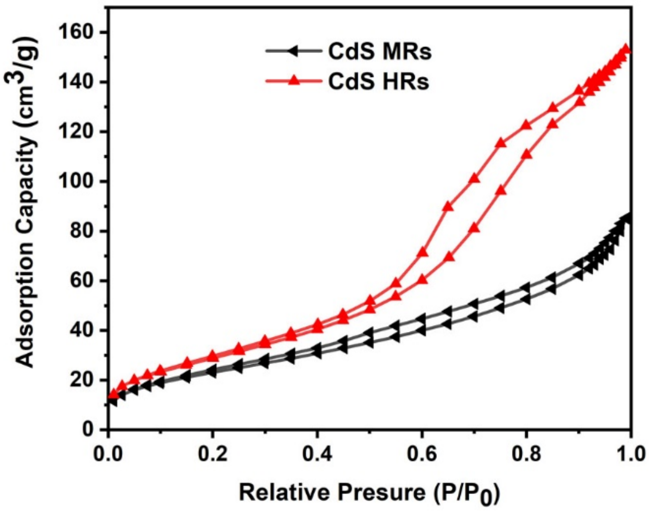

Figure S4: N_2 adsorption-desorption isotherms for CdS MRs and CdS HRs.

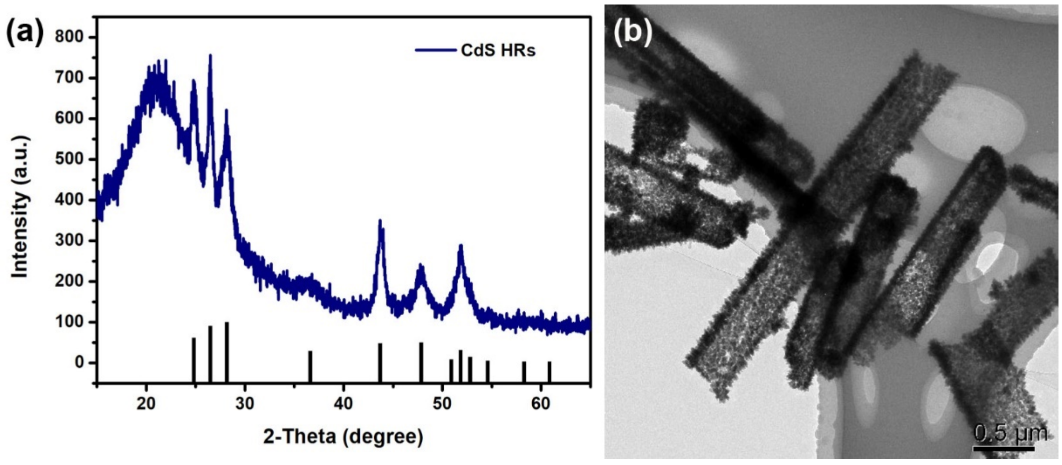

Figure S5: (a) XRD pattern and (b) TEM image of the CdS HRs after the cycling tests.
